# Quantitative Echotextural Attributes of Pectoralis Major Muscles in Broiler Chickens: Physicochemical Correlates and Effects of Dietary Fat Source

**DOI:** 10.3390/ani9060306

**Published:** 2019-05-31

**Authors:** Tomasz Schwarz, Katarzyna Połtowicz, Joanna Nowak, Maciej Murawski, Martyna M. Małopolska, Krzysztof Andres, Dorota Wojtysiak, Mark Jamieson, Pawel M. Bartlewski

**Affiliations:** 1 Department of Swine and Small Animal Breeding, University of Agriculture in Kraków, 24/28 Mickiewicza Ave., 30-059 Cracow, Poland; k.andres@ur.krakow.pl; 2 Department of Poultry Breeding, National Research Institute of Animal Production, 32-083 Balice, Poland; katarzyna.poltowicz@izoo.krakow.pl (K.P.); joanna.nowak@izoo.krakow.pl (J.N.); 3 Department of Animal Biotechnology, University of Agriculture in Kraków, 1B Rędzina St., 31-274 Cracow, Poland; rzmmuraw@cyf-kr.edu.pl; 4 Department of Pig Breeding, National Research Institute of Animal Production, Krakowska 1, 32-083 Balice, Poland; martyna.malopolska@izoo.krakow.pl; 5 Department of Animal Reproduction and Anatomy, 24/28 Mickiewicza Ave., 30-059 Cracow, Poland; d.wojtysiak@ur.krakow.pl; 6 Department of Biomedical Sciences, Ontario Veterinary College, University of Guelph, 50 Stone Rd., Guelph, ON N1G 2W1, Canada; mjamie02@uoguelph.ca (M.J.); pmbart@uoguelph.ca (P.M.B.)

**Keywords:** broiler chicken, pectoral muscle, ultrasonography, echotexture, physicochemical characteristics

## Abstract

**Simple Summary:**

There is presently no method of predicting meat quality based on a single examination of birds before slaughter. That could potentially be accomplished with a computer-assisted analysis of ultrasonographic images. Hence, this experiment was designed to draw correlations between various physical and chemical properties of chicken pectoral muscles and pixel values (brightness elements) measured in corresponding ultrasonograms (a.k.a. echotexture). Our study revealed that echotextural characteristics of pectoral muscles in live birds were predictive of several important physical parameters (e.g., cutting force, hardness, and chewiness) as well as intramuscular fat and protein content. However, different dietary fats that caused changes in the chemical composition of chicken breast also affected the associations between muscle echotexture and its physicochemical properties. For example, no correlations with the chemical composition of the muscles could be found in birds fed with soybean oil. We concluded that ultrasonographic imaging combined with a computerized image analysis can offer significant benefits to the poultry industry and consumers. It can aid in livestock genetic selection and improvement programs as well as enhance the quality of poultry meat and meat products. However, more confirmatory studies are needed.

**Abstract:**

This study examined the relationships among physicochemical properties and ultrasonographic image attributes of pectoralis major muscles in broiler chickens. Forty male Ross 308 chicks were randomly assigned to four equinumerous fat-supplementation groups (Group SO: soybean oil; Group FO: flax oil; Group SO + FO: soybean oil + flax oil; and Group BF: beef fat). Ultrasonograms of birds’ pectoral muscles were obtained just before slaughter at 6 weeks of age and were subjected to digital image analyses to determine the mean pixel intensity (MPI) and pixel heterogeneity values (standard deviation of numerical pixel values; MPH). A total of 2, 4, 2, and 6 significant correlations were recorded in Groups SO, FO, SO + FO, and BF, respectively; there were no correlations with the chemical composition of the muscles in Groups SO and SO + FO. The strongest correlations were found between muscle lightness (L*) and MPH in Group BF (physical characteristic; r = −0.82, *p* = 0.003), and between crude fat/protein content and MPI/MPH of pectoral the major muscles in Groups FO/BF (chemical characteristics; r = 0.72, *p* = 0.02). There exists a potential application of ultrasonographic imaging and computerized image analysis for predicting certain physicochemical properties of pectoralis major muscles in broiler chickens.

## 1. Introduction

In order to provide high quality products to consumers, it is necessary for the food industry to obtain reliable information on factors influencing meat quality throughout the entire production cycle [1]. In fact, both the quality control in commercial farms as well as the continued genetic selection in modern poultry operations requires a constant monitoring of meat productivity traits. At present, the vast majority of such traits are typically determined post mortem, which obviously precludes the optimal use of at least some of the superior animals for reproduction [2]. Several growth characteristics are simply not monitored regularly or are not determined at all because their assessment is too cumbersome or costly. Such parameters include an array of physicochemical properties of skeletal muscles and intramuscular fat (e.g., color, pH, compactness, and cutting force) that ultimately determine meat sensory characteristics [3,4]. There is currently no method of predicting the quality of meat based on a single examination of birds’ muscles before slaughter. Therefore, one of the challenges facing the modern poultry industry is to develop accurate, non-invasive, and inexpensive methods of predicting meat quality from observations and testing of live birds. 

It is feasible that all major physicochemical properties of muscles could be assessed using ultrasound technology. A promising new area of research that has not been fully explored yet is the potential for utilizing computer-assisted analyses of ultrasonographic images in situ for the prediction of tissues’ chemical compositions and histomorphological attributes [5,6,7,8]. Ultrasonographic transducers contain piezoelectric crystals, which upon application of the electrical current undergo structural deformation and emit high-frequency ultrasound beams [9,10]. When the ultrasound waves encounter the target tissue, they are transmitted through, absorbed, reflected, or scattered and the proportions of reflected and non-reflected waves determine the appearance of resultant ultrasonograms [9,10]. Ultrasound images consist of numerous pixels (or picture brightness elements) with the numerical values ranging from 0 (absolute black) to 255 (absolute white), and different types of tissues exhibit distinctive echotexture. Skeletal muscles have a heterogeneous echotexture; proteins generally absorb ultrasound waves and appear as dark areas, but the supportive structures that surround each muscle fiber and form fascicles exhibit anisotropy and deflect the ultrasound waves depending on the angle at which they are hit, thereby appearing as brighter spots [9,10]. The lipid content of tissues contributes significantly to their overall echointensity as fat is highly echogenic.

In the field of medicine, numerous studies have attempted to analyze tissue microstructure and chemical composition with ultrasonographic techniques. For instance, Clague et al. [11] reported that the quantitative ultrasonographic analysis could detect changes in the chemical composition of dystrophic muscles that were not detectable with computed tomography (CT) scans. In the field of animal and veterinary sciences, significant correlations have been found between changes in the chemical composition of ram testes and their echotextural characteristics [8]. These findings suggest that ultrasonography could indeed be used to assess histochemical changes in various internal organs and tissues, which leads to the concept that the quantitative ultrasonographic analyses could be useful indicators of the physicochemical characteristics of skeletal muscles.

We hypothesized that echotextural attributes of the pectoralis major muscle (M. pectoralis superficialis) in broiler chickens in situ would be significant predictors of its physicochemical properties. The specific objectives of the present study were (i) to examine pectoralis major muscles for correlations between their quantitative echotextural attributes and physicochemical properties and (ii) to assess the influence of the dietary fat source (i.e., single or mixed oils of plant origin and fat of animal origin) on the relationships between the echotexture, physical characteristics, and chemical composition of pectoralis major muscles in broiler chickens.

## 2. Materials and Methods 

### 2.1. Animals and Housing Conditions

The local Ethics Committee for Animal Experimentation approved all experimental procedures performed in the present study. This experiment utilized forty 42-day-old male broiler chicks (Ross 308). One-day-old chickens hatched in a commercial poultry hatchery were transported to an experimental farm of the National Research Institute of Animal Production in Aleksandrowice near Cracow, Poland. The birds were randomly assigned to four experimental groups based on the source of fat in the feed mixtures: soybean oil (Group SO), flaxseed oil (Group FO), mixed oil (Group SO + FO; in proportion 56%:44% of soybean and flaxseed oil), or beef fat (Group BF). Broilers were kept in pens on deep litter in automatically controlled environmental conditions. An initial temperature was set at 30 °C and was decreased gradually to 20 °C until the end of the experiment. Lighting was provided for 23 h per day (1−7 days), 20 h per day (8−39 days), and 23 h per day (40−42 days). The birds were fed ad libitum with complete starter (1−21 days), grower (22−35 days), and finisher (36−42 days of age) mixtures containing 22%, 20.5%, and 20.5% crude protein and 2990, 3130, and 3130 kcal/kg metabolizable energy, respectively, and had unrestricted access to drinking water. Each group of birds received feed mixtures that differed only in the dietary fat source, and the net fat contents in the consecutive diets (starter, grower, and finisher) were 2.9%, 4.8%, and 4.8%, respectively. The mixtures were composed according to the dietary requirements for meat-type chickens [12]. The chickens were reared to 42 days of age at which time their mean (± SEM) body weight was 2.98 ± 0.05kg; all birds were then sacrificed by decapitation after 10 h of fasting.

### 2.2. Sampling and Laboratory (Physicochemical) Analyses

The pH values of the pectoralis major muscles were determined 15 min post mortem and after 24 h of air-chilling at 4 °C to examine the rate and intensity of muscle acidification, using a portable CyberScan10 pH meter (Eutech Instruments Pte Ltd., Singapore) equipped with a glass electrode and a temperature probe for automatic temperature adjustments. The pH meter had been calibrated using two calibration buffers (pH 4.01 and pH 7.00) at a temperature similar to the temperature of muscles, and then, measurements were taken by placing the electrode at a 45° angle mid-way through the body of the muscle.

The color of the inner surface of each muscle was determined immediately after a dissection using the reflectance spectrophotometer Minolta CR 310 Chroma Meter (Konica Minolta Sensing Business Unit, Osaka, Japan) equipped with a 50-mm reading head. Whenever possible, the entire surface area of the muscle was analyzed; however, a special care was taken to avoid areas containing visible bruises, hemorrhages, or superficial blood vessels [1]. Four consecutive measurements were taken to calculate the mean values for muscle lightness (L*), color saturation towards red (a positive a* value), and color saturation towards yellow (a positive b* value), according to the L* a* b* scale [13].

The amount of exudate from the pectoralis major muscles was measured 48 h post mortem for the determination of their drip loss. Meat samples (weighing ~80 g) were placed in tightly sealed containers and stored in a refrigerator at 4 °C. Drip loss was expressed as the percentage of sample weight loss in relation to its weight recorded before refrigeration. The thawing weight loss was determined after 28 days of storage at ~18 °C. Samples of the muscles weighing ~80 g were stored in sealed containers and then moved to a cold storage room, where they were thawed for 24 h at 4 °C. After thawing, the samples were re-weighed, and the weight loss was expressed as the percentage of initial (pre-freezing) weight. The cooking weight loss was defined as the percentage loss of meat weight after cooking in a water bath at 100 °C until the attainment of a core temperature of 76 °C (measured in the thickest portion of the sample). All samples (~80 g each) were cooked individually in small plastic bags, then chilled at room temperature for 30 min, and finally kept at 4 °C for 45 min before determining the relative thermal loss (expressed as the percentage of initial, precooking weight).

After weighing for the determination of thermal loss, the chilled meat samples were prepared for shear force and texture measurements. Samples measuring ~1.3 cm in diameter and ~3 cm (for shear force determination) or ~1.5 cm in length (for texture analysis) were removed from each sample parallel to the fiber orientation through the thickest portion of the cooked muscle. The Warner–Bratzler shear force was determined using an Instron 5542 system (Instron^®^, High Wycombe, UK) as the maximum force (N) perpendicular to the fibers. A texture analysis, which included hardness (N), springiness (mm), elasticity, cohesiveness, gumminess (N), and chewiness (MJ), was performed with the same instrument fitted with a 50-mm compression attachment using a Texture Profile Analysis (TPA) software (Instron^®^, High Wycombe, UK). A double compression test with a 60% deformation was performed with a crosshead speed of 200 mm/min, an interval of 2 s between successive compressions, and detection threshold of 0.01 N. The samples were compressed perpendicularly to the muscle fiber orientation. All physicochemical analyses of the broiler chicken breast muscles were conducted in the National Research Institute of Animal Production.

### 2.3. Measurement of Muscle Fiber Diameter and Chemical Analyses

Within 15 min post mortem, a block measuring ~20 mm in length and with a base area of ~100 mm^2^ (obtained parallel to visible muscle fibers in the central portion of the muscle) was cut out of the left pectoralis major; frozen in isopentane, which was precooled with liquid nitrogen; and then stored at ~80 °C until the histological examinations at a later date. The tissue samples were mounted on a cryostat chuck with a few drops of Tissue-Tek freezing medium (Sakura Finetek Europe, Zoeterwoude, Holland). Transverse sections (10 µm in thickness) were cut at ~20 °C with the cryostat (Slee MEV, Mainz, Germany) and stained with hematoxylin and eosin. The sections were then dehydrated in a graded series of ethyl alcohol, cleared in xylene, and mounted onto the glass slides using DPX mounting medium (Fluka Chemie GmbH, Buchs, Switzerland). A minimum of 200 fibers was counted in each section using a Nikon E600 (Nikon Ltd., Tokyo, Japan) light microscope. The diameters of the muscle fibers were measured with the image analytical software MultiScan^®^14.02 (CSS Ltd., Warsaw, Poland). Samples of the breast (pectoralis major muscle) were collected from birds of each group in order to determine the chemical composition, i.e., the contents of total dry matter, total protein (Kjeldahl method), and crude fat (Soxhlet method) using the AOAC standards [14].

### 2.4. Ultrasonography and Echotextural Analyses

**Figure 1:**
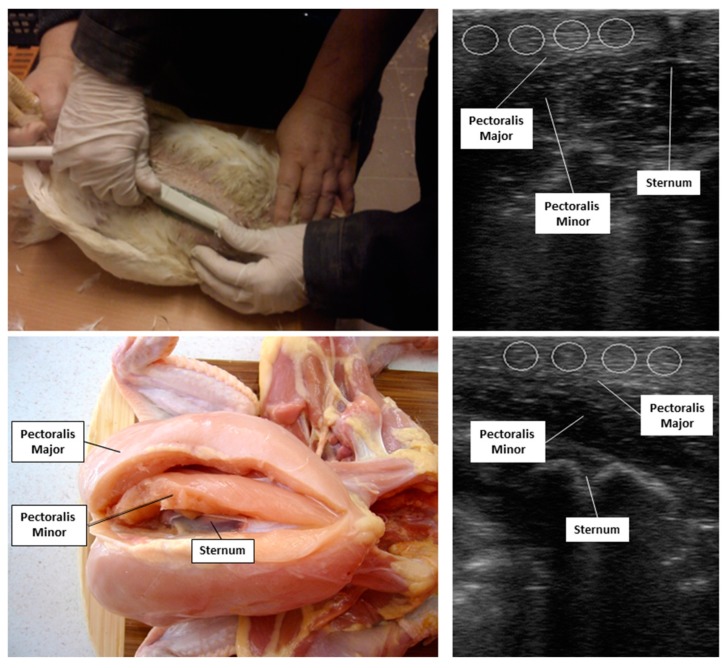
In situ ultrasonographic examinations (upper left) of pectoralis major muscles (bottom left—https://kriegerscience.wordpress.com/2010/11/09/dissecting-a-whole-chicken/) in broiler chickens (n = 40) scanned in a transverse (upper right) or longitudinal (bottom right) plane: The white circles in the panels depicting ultrasonograms indicate the potential placement of four computer-generated circular areas of interest (spot meters) used for echotextural analyses of the muscle tissue.

Ultrasonograms of the birds’ pectoralis major muscles were obtained just before slaughter, using the Aloka PS2 ultrasound scanner (Aloka Ltd., Tokyo, Japan) connected to a hand-held 5.0-MHz linear-array transducer (Figure 1). Each muscle was scanned in a transverse and longitudinal plane, and still images containing the largest cross-sectional areas of the muscle were saved as digital (DICOM) images with the resolution of 640 × 480 pixels. All echotextural analyses were subsequently conducted at the University of Guelph, ON, Canada and utilized the ImageProPlus^®^7.0 analytical software (Media Cybernetics Inc., Rockville, MD, USA). Four identical, non-overlapping circular spot meters (33 pixels in diameter) were used to compute the mean pixel intensity (MPI) and mean pixel heterogeneity (or standard deviations of the mean pixel values-MPH) of each ultrasonogram; the echotextural variables for all spot meters were then averaged to give the final echotextural value of a muscle.

### 2.5. Statistical Analyses

One-way analysis of variance (ANOVA) and Tukey tests were used to compare the weights of birds/carcasses and the physicochemical variables of pectoralis major muscles among the four groups of broiler chickens studied (SigmaPlot^®^11.0; Systat Software Inc., San Jose, CA, USA). Echotextural differences between the groups and scanning planes (transverse vs. longitudinal) were analyzed by two-way ANOVA and the least significant difference (LSD) tests for comparisons of the individual means. A Pearson product moment analysis was used to examine the correlations among quantitative echotextural attributes and the physical properties/chemical composition of pectoralis major muscles for the four fat-supplementation groups of birds. Since there were no significant differences in echotextural variables between the lateral and transverse scanning planes, the data averaged on a per animal basis were used for correlation analyses. A *p* value ≥ 0.05 was considered statistically significant, and all results are given as the mean ± standard error of the mean (SEM).

## 3. Results

### 3.1. Weight of Birds and Physicochemical Characteristics of Pectoral Muscles

The mean body weight of the chickens before slaughter was greatest (*p* < 0.05) in the mixed-oil group (Group SO + FO; 3057 ± 5 g), and it was greater (*p* < 0.05) for Group BF (3012 ± 5 g) compared with Groups SO (2899 ± 6 g) and FO (2912 ± 4 g) of broiler chickens. The mean weight of chilled carcasses was greatest (*p* < 0.05) for Group SO + FO birds (2460 ± 12 g) followed by Groups BF (2349 ± 16 g) and FO (2346 ± 10 g), and it was the lowest (*p* < 0.05) for Group SO (2266 ± 12 g). There were no significant differences among the four fat-supplementation groups of chickens in the mean estimated weight of combined pectoral muscles (674 ± 14 g).

The pH of the muscles recorded 24 h post mortem were significantly lower in Group FO and Group SO + FO compared with Group BF (Table 1). The color saturation towards yellow (b*) was significantly greater in Group SO + FO than in Group BF birds. In addition, the percentage weight loss post-thawing was significantly greater in Group FO compared with Group BF, and the total thermal loss was significantly greater in Group SO compared with Group BF chickens. No other physical variables differed among the four groups of broiler chickens studied (*p* > 0.05). The total protein content was greater (*p* < 0.05) in Group BF compared with Group SO, but both Groups SO and FO exceeded (*p* < 0.05) Group BF in crude fat content of the pectoralis major muscles.

### 3.2. Differences in Echotextural Variables

There was a significant main effect of the fat-supplementation group for the mean pixel intensity (MPI) of pectoralis major muscles in the birds of the present study (Table 2). The MPI of the muscles in Group FO broiler chickens was significantly greater than that in Group BF birds. Group SO birds exceeded (*p* < 0.05) Group BF animals in mean MPI values of the left pectoralis major muscle scanned in the transverse plane.

There was no significant main effect of either dietary group or scanning plane for MPH values determined in the ultrasonograms of pectoralis major muscles, but the interaction of the two terms was statistically significant. Longitudinal scans of the left pectoralis major muscle in Group FO chickens exhibited a greater (*p* < 0.05) MPH than those of the right muscle obtained in both scanning planes. In addition, the MPH of the left pectoralis major muscle in the longitudinal plane was less (*p* < 0.05) in Group SO compared with Groups FO, SO + FO, and BF.

### 3.3. Correlation Analyses

Significant correlations between the echotextural variables and physicochemical characteristics of the pectoralis major muscle determined in the present study are summarized in Table 3. With data analyzed for each treatment group separately, a total of 14 correlations were recorded between ultrasonographic image attributes and physicochemical parameters; eleven correlations were found with physical properties and three correlations with chemical properties. There were seven correlations between MPI and physiochemical properties and seven correlations between MPH and physicochemical properties of the muscles. Within the four treatment groups, two correlations were found in Group SO (MPI-1 and MPH-1), four correlations in Group FO (MPI-4), two correlations in Group SO + FO (MPI-2), and six correlations in Group BF (MPH-6). pH_15 min_, cutting force, color saturation towards yellow (b*), and crude fat content exhibited two significant correlations each (pH_15 min_: with MPH in Group SO and BF; cutting force: with MPI in Group FO and with MPI in Group SO+FO; color saturation towards yellow (b*): with MPI in Group FO and with MPH in Group BF; and crude fat: with MPI in Group FO and with MPH in Group BF). With respect to the relative sensitivity of ultrasound imaging, muscle lightness (L*) was the variable with the strongest correlations to image attributes (with MPI Group BF; r = −0.82; *p*= 0.003). The strongest correlations for each group of birds were as follows: Group SO—between MPH and pH_15 min_ (r = −0.72, *p* = 0.02); Group FO—between MPI and color saturation towards yellow (b*) (r = −0.70, *p* = 0.02); Group SO+FO—between MPI and hardness (r = −0.69, *p* = 0.03); and Group BF—between MPH and lightness (L*). When data were pooled and analyzed for all animals, there were only three significant overall correlations (between MPI values and gumminess, chewiness, and crude fat content of pectoral muscles).

## 4. Discussion

The pectoralis major muscles of Group SO and Group FO birds had significantly higher crude fat and lower crude protein contents compared with Group BF. This result contrasts with the findings of a previous study by Cortinas et al. [15], which demonstrated that increasing polyunsaturated fatty acids (PUFAs) in diet by 46 g/kg decreased crude fat in chicken thigh meat by ~17%. However, the accumulation of lipids in leg muscles of broiler chickens is generally greater compared with that of breast muscles. In another study, replacing beef fat with soybean oil and flax oil also decreased fat deposition in broiler chickens [16]. The reasons for discrepancies between those earlier and our present study are difficult to explain. It is possible, however, that in broiler chickens allocated to both the soybean oil- and flaxseed oil-supplemented groups, there was a decreased fat deposition but even more decreased amino acid uptake by growing muscles. Consequently, this could have resulted in a higher percentage of muscular crude fat even though the absolute amount of fat was less in the muscles of Group SO and FO birds compared with Group BF animals. The pH of pectoralis major muscles was significantly lower 24 h post mortem in both flax oil-supplemented groups (Group FO and SO + FO) compared with Group BF, but such a difference in pH values was not observed 15 min post mortem. This is consistent with a study by Simmons et al. [17] who demonstrated that a flaxseed oil-enhanced diet gradually decreased the pH in Brook trout fillets over a period of 11 days although no difference in pH was observed in the fillets immediately after slaughter. Muscle pH post mortem decreases due to the cleavage of muscle proteins into smaller peptide chains, free amino acids, and ammonia [17]. Thus, the present results may be interpreted to suggest that flaxseed oil-supplemented diets increased the rate of cleavage of muscle proteins, resulting in a lower muscle pH. Alternatively, flaxseed oil may have induced a decline in pH through an increase in carbonic acid formation; Jezek and Buchtova [18] demonstrated that CO_2_ diffusion, which parallels the formation of carbonic acid, decreased muscle pH in carp over a 15-day period. Janisch et al. [3] observed that the post mortem pH was negatively associated with b* values in the pectoral muscles of broiler chickens. This finding is in agreement with the present results as a low acidic pH in Group MO was associated with a high b* whereas a relatively high acidic pH was associated with the lowest b* value in Group BF. The post-thawing weight loss was greater in Group FO, and the total thermal loss was greater in Group SO compared with Group BF. In both instances, the intermediate values for Group SO + FO did not vary from all other groups. These results suggest that, with regards to the thawing-induced and total thermal weight losses, combining both soybean oil and flaxseed oil supplementation had a negating effect on the impact of individual plant oil supplements. 

There were differences among the four treatment groups of birds in both of the echotextural variables of the left pectoralis major muscle (MPI in the transverse plane and MPH in the longitudinal plane). In addition, the mean MPH of the left pectoralis major muscle in the transverse plane was greater than the pixel heterogeneity of the right muscle in either the transverse or longitudinal plane. Differences among the four fat-supplementation groups may be due to a summation of differences in their physicochemical properties that were more pronounced in the left compared with the right pectoral muscle. Similarly, the differences in MPH between the right and left muscles and scanning planes may be indicative of the varying chemical composition or histomorphological properties [6,8]. Nielson et al. [19] observed a lower in the pixel intensity of transverse scans compared with longitudinal scans of the supraspinatus and vastus lateralis muscles. In contrast, Reimers et al. [20] reported greater echogenicity in the transverse compared with longitudinal scans of various skeletal muscles in humans. The histological and physicochemical properties of pectoralis major muscles in broiler chickens vary depending on intramuscular locations; the fiber diameters of pectoral major muscle are significantly smaller in the anterior and middle regions in comparison to the posterior region of the muscle [21]. However, there were no correlations between echotextural attributes and muscle fiber diameters in this study. Therefore, it is logical to infer that differences in echotextural attributes between scanning planes were due mainly to variations in the chemical constituents of the muscle associated with their non-uniform intramuscular distribution. More studies are needed in that area.

No previous studies have investigated the relationship between echotextural attributes of skeletal muscles and their physical properties. In the present study, there were only two instances of correlations for the same echotextural and physical variable recorded in more than one group of broiler chickens (between MPI and cutting force in Groups FO and SO + FO and between MPH and pH_15 min_ in Groups SO and BF). Moreover, even though correlations between MPI and cutting force were of identical magnitude, they were in opposite directions (an inverse relation in Group FO and a positive correlation in the mixed plant oil group; Table 3). Connective tissues play an important role in determining meat quality; the perimysium surrounding muscle fascicles contributes to the overall echogenicity of the muscle and creates a specked appearance in ultrasound images [20]. Clearly, the determinants of the cutting force, such as relative amounts of connective tissue [2], may be responsible for the interactions with ultrasound waves that impinge on muscular echotexture. Nevertheless, the specific reasons for the opposite correlational directions in Groups FO and SO + FO remain unknown. The remaining significant correlations among echotextural and physical properties of the pectoral major muscles of broiler chickens were equally inconsistent across the four dietary groups.

As with the physical properties above, there was a lack of consistency in correlations among the echotextural attributes and chemical composition of pectoralis major muscles. A positive correlation was recorded between MPI and crude fat content of the muscles in Group FO and a negative correlation between MPH and crude fat content in Group BF. In rams’ testes, pixel heterogeneity correlated directly with extractable lipids [8]. A positive correlation between MPH and crude protein content was observed in Group BF. Proteins are generally thought to attenuate ultrasound waves [22], and previous studies have shown that the body of the muscle in ultrasound images appears dark relative to fat or dense connective tissues such as muscle epimysium [18,19]. However, Ahmadi et al. [8] reported a negative correlation between MPH and the crude protein content in ram testes. These results may be explained by the difference in protein makeup of testes and skeletal muscles. A large proportion of proteins in testicular tissue are membrane and globular proteins [23], whereas much of the muscle protein is in the form of myofibrillar protein, which is less soluble than sarcoplasmic proteins [19]. Connective tissues, such as collagen, also contribute to crude protein content of the muscle [24]. Even though a computer-assisted image analysis only selects the internal portion of the muscle, which excludes superficial connective tissues (i.e., muscle epimysium), external layers may significantly alter the image characteristics (i.e., numerical pixel values of ultrasonograms); an attenuation of ultrasound waves by the scrotal skin and tunica vaginalis significantly changed the echotexture characteristics of testicular parenchyma [7].

There was a complete lack of correlations among the ultrasonographic characteristics and chemical composition of pectoralis major muscles in Group SO and SO + FO. It is attractive to speculate that the absence of correlations in those groups may be due to specific ultrasonographic properties of soybean oil precluding our being able to detect correlations with any chemical constituent in the pectoralis major muscles of broiler chickens studied. It is feasible that MPI values may only be used to predict the content of chemical constituents that comprise a sufficient amount of the muscle tissue (i.e., equal to or greater than the “threshold” level). A correlation between crude fat and MPI was recorded in Group FO, which had the highest crude fat content. Moreover, the threshold content may vary between proteins and lipids. In the present study, the crude fat content of pectoralis major muscles was approximately ~2%, while the crude protein content was ~23%. Lipids significantly increase tissue echointensity, and so, it is attractive to assume that a 10-fold lower amount of fat compared with proteins is required for the detection of quantitative correlations with numerical pixel values. As with the MPI values discussed above, ultrasound imaging may require a minimum degree of heterogeneity in the muscle tissues to detect correlations between MPH and chemical constituents. The pectoral muscles from Group BF chickens exhibited the highest crude protein and lowest crude fat content; this contrast is likely the reason why Group BF had the greatest MPH values and why MPH, in this subset of animals, could be large enough to identify correlations with crude protein and crude fat content.

The pectoral muscles of broiler chickens in this study were examined with a 5.0-MHz transducer. Earlier ultrasonographic studies of skeletal muscles utilized ultrasound frequencies ranging from 5 to 8 MHz. High-frequency waves are suitable for imaging mainly superficial structures as they are more attenuated than lower-frequency waves for a given distance, but they offer images of higher resolution [25,26]. The influence of the scanning frequency on quantitative relationships between the echotextural attributes and chemical composition of various tissues and organs has not been sufficiently explored. 

## 5. Conclusions

To recapitulate, ultrasonographic image attributes were significantly correlated with certain physicochemical properties of broiler chicken pectoralis major muscles. Different dietary fats induced significant differences in physicochemical characteristics of the muscles among the four fat-supplementation groups of birds. The present study demonstrates the potential applicability of computerized analysis of ultrasonograms to study certain physicochemical characteristics of pectoral major muscles in broiler chickens. At present, however, due to the lack of consistent correlations across the dietary groups and considering significant differences in echotextural parameters between the scanning planes or right and left pectoralis major muscles, we remain skeptical of the immediate utility of this technique for predicting physiochemical properties of the muscles in commercial settings, at least until further confirmatory studies are complete.

## Figures and Tables

**Table 1:** The mean (± SEM) fiber diameters and physicochemical properties of pectoralis major muscles in broiler chickens allocated to four fat-supplementation groups (each n = 10): Within the rows, mean values denoted by different letters are significantly different (*p* < 0.05).

Variable/Group	Group SO Soybean Oil	Group FO Flax Oil	Group SO + FO Soybean + Flax Oil	Group BF Beef Fat
Fiber diameter (µm)	59.2 ± 1.9	60.5 ± 1.1	59.7 ± 1.4	59.2 ± 1.2
pH_15 min_^1^	6.55 ± 0.05	6.67 ± 0.05	6.60 ± 0.04	6.60 ± 0.06
pH_24 h_^2^	6.29 ± 0.03ab	6.20 ± 0.04a	6.20 ± 0.03a	6.35 ± 0.04b
Lightness (L*)	53.8 ± 0.5	54.8 ± 0.7	54.6 ± 1.0	55.4 ± 0.8
Color channel a*	11.5 ± 0.3	11.0 ± 0.3	10.9 ± 0.4	10.3 ± 0.2
Color channel b*	12.7 ± 0.5ab	12.5 ± 0.4ab	14.0 ± 0.8a	11.0 ± 0.3b
Exudate 48 h	1.12 ± 0.15	0.98 ± 0.12	0.90 ± 0.13	0.84 ± 0.12
Exudate 48 h right (intact)	1.22 ± 0.20	0.83 ± 0.07	0.93 ± 0.14	0.74 ± 0.10
Exudate 48 h left (cut)	1.03 ± 0.10	1.14 ± 0.20	0.87 ± 0.12	0.94 ± 0.15
% weight loss post-thawing (right + left)	2.9 ± 0.2ab	3.4 ± 0.4a	2.5 ± 0.4ab	2.2 ± 0.3b
% weight loss post-thawing (right)	3.6 ± 0.4	3.6 ± 0.4	2.2 ± 0.4	2.1 ± 0.4
% weight loss post-thawing (left)	2.2 ± 0.22	3.1 ± 0.5	2.9 ± 0.5	2.2 ± 0.4
Total thermal loss (%)	24.2 ± 0.9a	22.5 ± 0.7ab	22.2 ± 0.6ab	21.2 ± 0.4b
Cutting force (N)	16.5 ± 0.9	16.2 ± 0.7	16.1 ± 0.9	16.0 ± 0.7
Hardness (N)	18.4 ± 0.6	18.6 ± 0.9	19.4 ± 0.7	18.3 ± 0.7
Springiness (mm)	3.06 ± 0.04	2.87 ± 0.07	3.29 ± 0.25	3.07 ± 0.04
Elasticity	0.80 ± 0.005	0.81 ± 0.004	0.80 ± 0.006	0.79 ± 0.007
Compactness	0.34 ± 0.008	0.35 ± 0.008	0.34 ± 0.007	0.33 ± 0.01
Gumminess (N)	6.3 ± 0.3	6.4 ± 0.3	6.7 ± 0.3	6.1 ± 0.4
Chewiness (mJ)	19.3 ± 1.0	18.5 ± 1.2	21.9 ± 1.8	19.0 ± 1.4
Dry matter (%)	24.18 ± 0.52	24.96 ± 0.34	24.89 ± 0.18	24.81 ± 0.17
Total protein (%)	22.17 ± 0.54a	22.60 ± 0.29ab	23.02 ± 0.16ab	23.57 ± 0.25b
Crude fat (%)	2.13 ± 0.10b	2.28 ± 0.20b	1.85 ± 0.11ab	1.55 ± 0.14a

^1^ pH measured 15 min after slaughter; ^2^ pH measured 24 h after slaughter.

**Table 2:** Echotextural characteristics of pectoralis major muscles in four fat-supplementation groups of Ross 308 broiler chickens (each n = 10). T: transverse plane; L: longitudinal plane; MPI: mean numerical pixel values (pixel intensity); and MPH: mean pixel heterogeneity (standard deviation of mean numerical pixel values). Means denoted by different letters are significantly different (*p* < 0.05); ab—within rows and AB—within columns.

Variable/Group	Group SO Soybean Oil	Group FO Flax Oil	Group SO + FO Soybean + Flax Oil	Group BF Beef Fat	Scanning Plane Mean
RightT-MPI	67.7 ± 2.3	66.8 ± 3.0	60.9 ± 2.9	60.7 ± 3.6	64.0 ± 1.5
RightL-MPI	67.2 ± 2.2	72.1 ± 2.4	66.6 ± 3.0	66.7 ± 3.3	68.1 ± 1.4
LeftT-MPI	69.5 ± 2.9a	66.8 ± 3.8ab	64.4 ± 3.2ab	56.3 ± 3.4b	64.3 ± 1.8
LeftL-MPI	63.6 ± 3.7	69.8 ± 3.3	66.7 ± 2.4	62.3 ± 2.4	65.6 ± 1.5
Group mean	67.0 ± 1.4ab	67.6 ± 2.0a	64.6 ± 1.4ab	61.5 ± 1.6b	-
RT-MPH	17.7 ± 0.7	17.4 ± 0.5B	17.9 ± 0.4	19.2 ± 0.4	18.1 ± 0.3
RL-MPH	18.6 ± 0.5	17.3 ± 0.5B	17.4 ± 0.5	18.1 ± 0.6	17.9 ± 0.3
LT-MPH	18.0 ± 0.7	18.6 ± 0.8AB	17.9 ± 0.5	17.1 ± 0.4	17.9 ± 0.3
LL-MPH	16.5 ± 0.9b	19.7 ± 0.6aA	19.1 ± 0.3a	18.9 ± 0.7a	18.6 ± 0.4
Group mean	17.7 ± 0.4	18.1 ± 0.4	18.1 ± 0.2	18.3 ± 0.3	-

**Table 3:** Summary of significant correlations between echotextural variables and physical properties or the chemical composition of the pectoralis major muscles for each group of broiler chickens receiving four different types of dietary fat.

Input Variable (x)	Output Variable (y)	r ^1^	*p* Value	Regression Equation
Group SO Soybean oil (n = 10)				
MPI ^2^	pH_24 h_^4^	0.64	0.05	y = 0.009x + 5.7
MPH ^3^	pH_15__min_^5^	−0.72	0.02	y = −0.07x + 7.8
Group FO Flax oil (n = 10)				
MPI	b*	−0.70	0.02	y = −0.12x + 20.9
MPI	Cutting force (N)	−0.64	0.04	y = −0.18x + 28.6
MPI	Elasticity	−0.67	0.03	y = −0.001x + 0.9
MPI	Crude fat (%)	0.72	0.02	y = 0.04x − 0.6
Group SO + FO Soybean + flax oil (n = 10)				
MPI	Cutting force (N)	0.64	0.05	y = 0.20x + 3.5
MPI	Hardness (N)	−0.69	0.03	y = −0.18x + 30.9
Group BF Beef fat (n = 10)				
MPH	pH_15 min_	−0.68	0.03	y = −0.09x + 8.3
MPH	L* ^6^	−0.82	0.003	y = −1.38x + 81.3
MPH	a* ^7^	0.64	0.05	y = 0.27x + 5.3
MPH	b* ^8^	−0.78	0.008	y = −0.42x + 18.8
MPH	Total protein (%)	0.72	0.02	y = 0.41x + 16.1
MPH	Crude fat (%)	−0.64	0.05	y = −0.20x + 5.2
Overall (n = 40)				
MPI	Gumminess (N)	−0.36	0.02	y = −0.04x + 9.2
MPI	Chewiness (mJ)	−0.31	0.05	y = −0.16x + 30.3
MPI	Crude fat (%)	0.53	0.0002	y = 0.03x − 0.004

^1^ coefficient of correlation; ^2^ mean pixel intensity; ^3^ mean pixel heterogeneity (standard deviation of numerical pixel values); ^4^ pH measured 24 h after slaughter; ^5^ pH measured 15 min after slaughter; ^6^ muscle lightness, and ^7^ color saturation towards red or ^8^ towards yellow.
